# Supplementary material for: Transcriptome-wide association analysis of brain structures yields insights into pleiotropy with complex neuropsychiatric traits
Source: Nat Commun. 2021 May 17;12:2878. doi: 10.1038/s41467-021-23130-y (PMC8128893; doi:10.1038/s41467-021-23130-y)
Supplement: Supplementary file 1 — Supplementary Information [file 41467_2021_23130_MOESM1_ESM.pdf]

# Supplementary information: Transcriptome-wide association analysis of brain structures yields insights into pleiotropy with complex neuropsychiatric traits

March 26, 2021

## 1 Supplementary figures

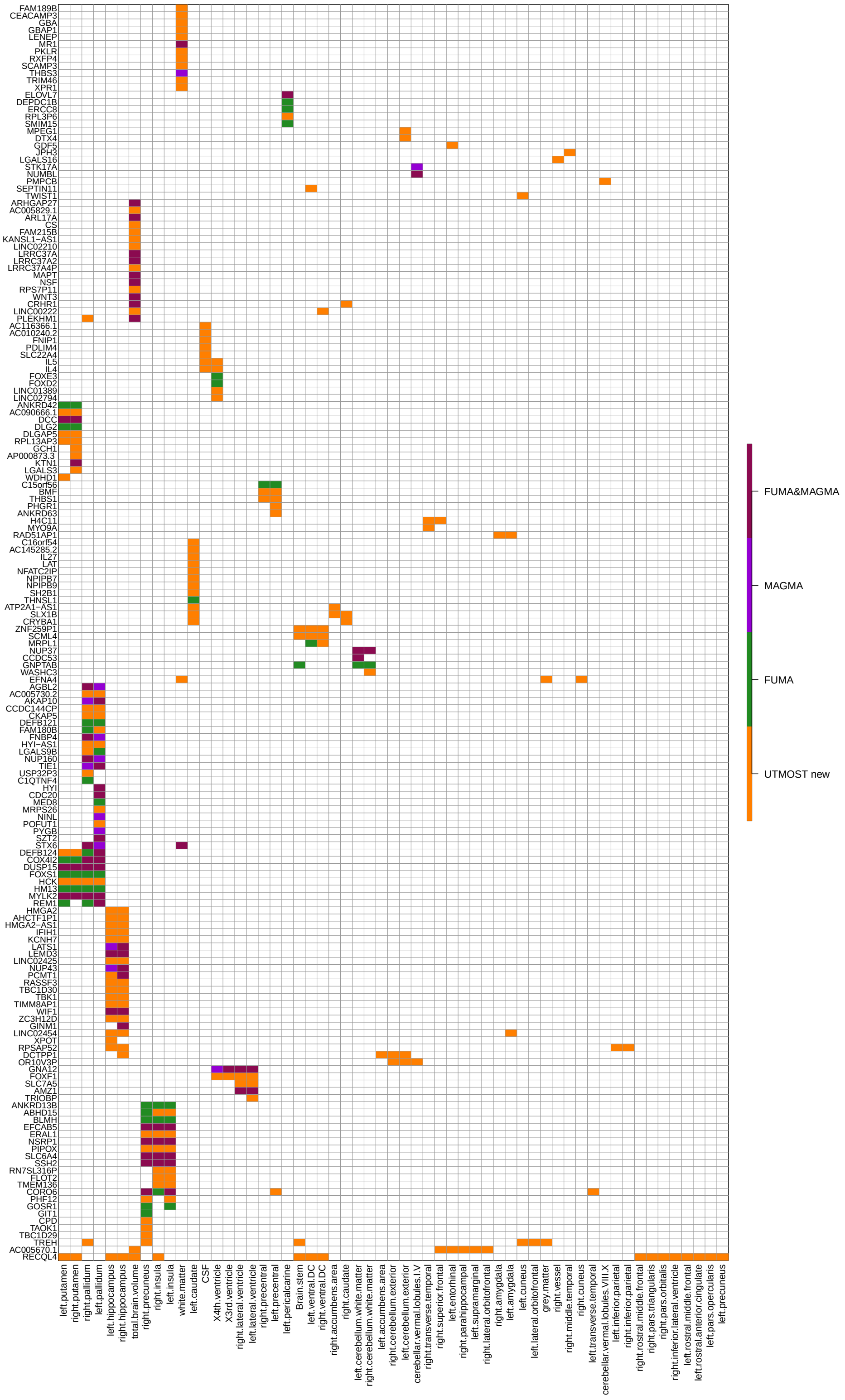


**Supplementary Figure 1: Significant gene-trait associations discovered in UKB cross-tissue TWAS analysis of ROI volumes (n=19,629 subjects).** FUMA: associations identified in FUMA; MAGMA: associations identified in MAGMA; FUMA&MAGMA: associations identified in both FUMA and MAGMA analysis; UTMOST new: novel associations identified in cross-tissue TWAS analysis.

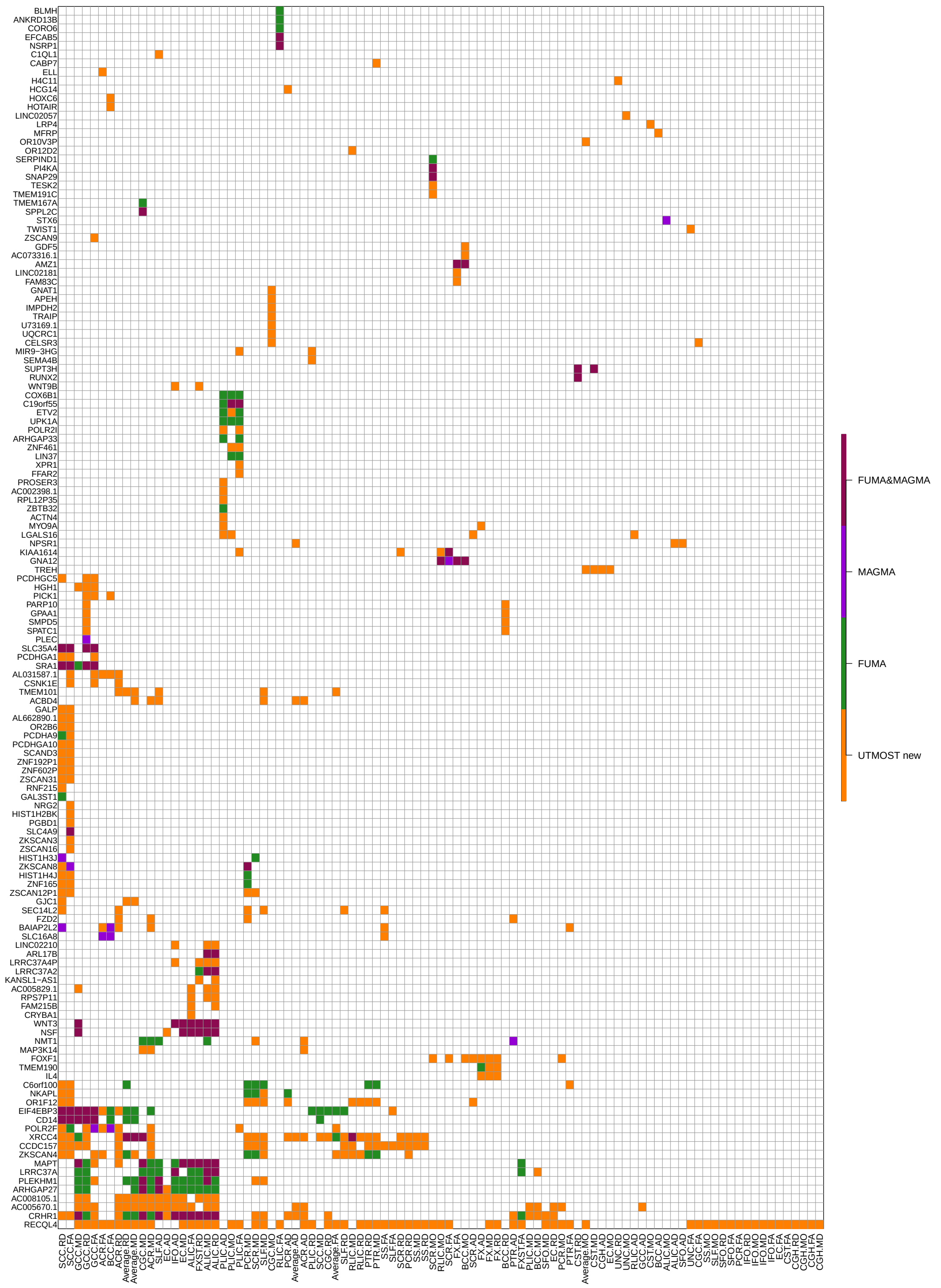


**Supplementary Figure 2: Significant gene-trait associations discovered in UKB cross-tissue TWAS analysis of DTI parameters (n=17,706 subjects).** FUMA: associations identified in FUMA; MAGMA: associations identified in MAGMA; FUMA&MAGMA: associations identified in both FUMA and MAGMA analysis; UTMOST new: novel associations identified in cross-tissue TWAS analysis.

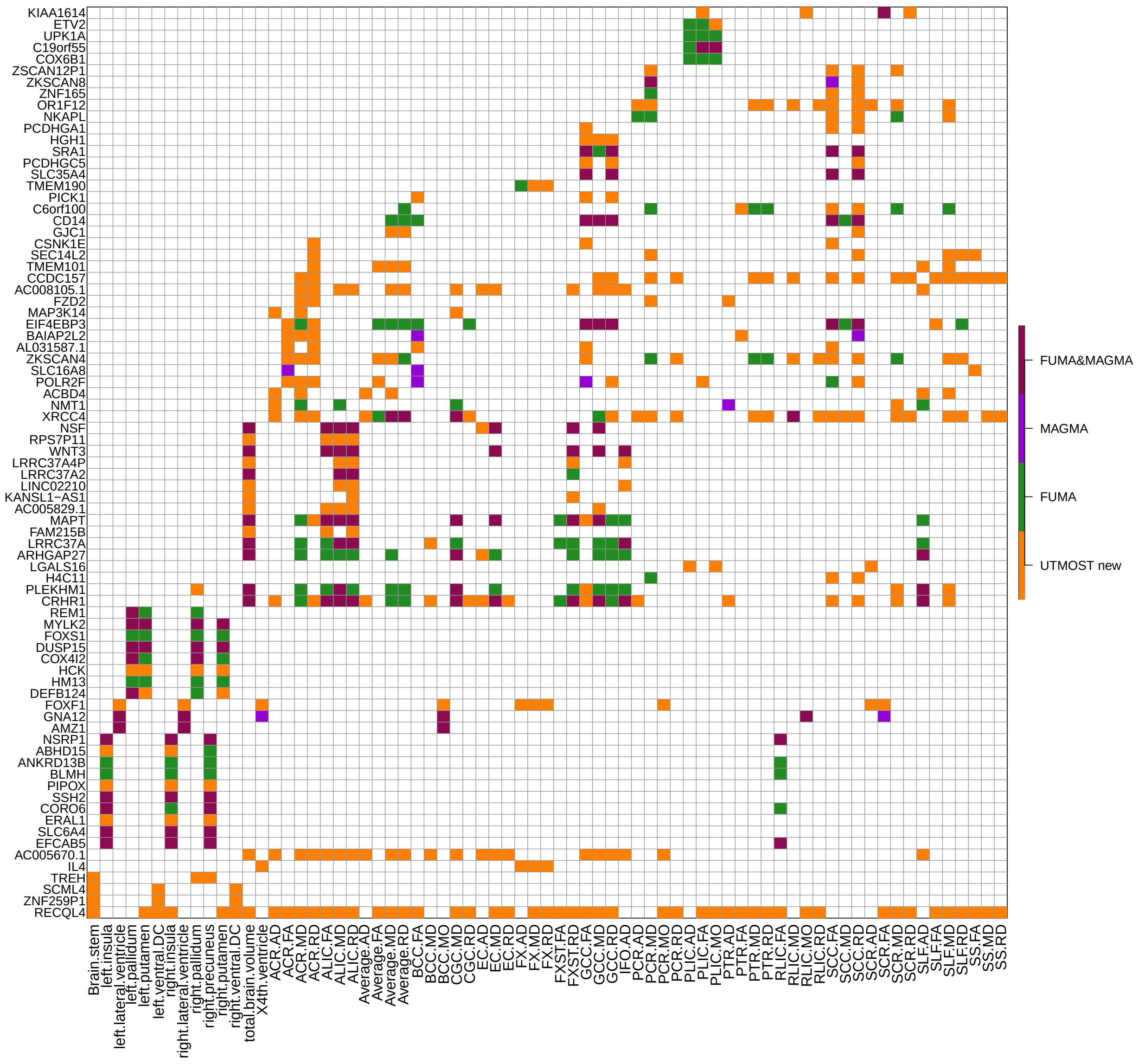


**Supplementary Figure 3: Selected significant gene-trait associations discovered in UKB cross-tissue TWAS analysis of 211 neuroimaging traits (n=19,629 subjects for ROI volumes and 17,706 for DTI parameters).** FUMA: associations identified in FUMA; MAGMA: associations identified in MAGMA; FUMA&MAGMA: associations identified in both FUMA and MAGMA analysis; UTMOST new: novel associations identified in cross-tissue TWAS analysis.

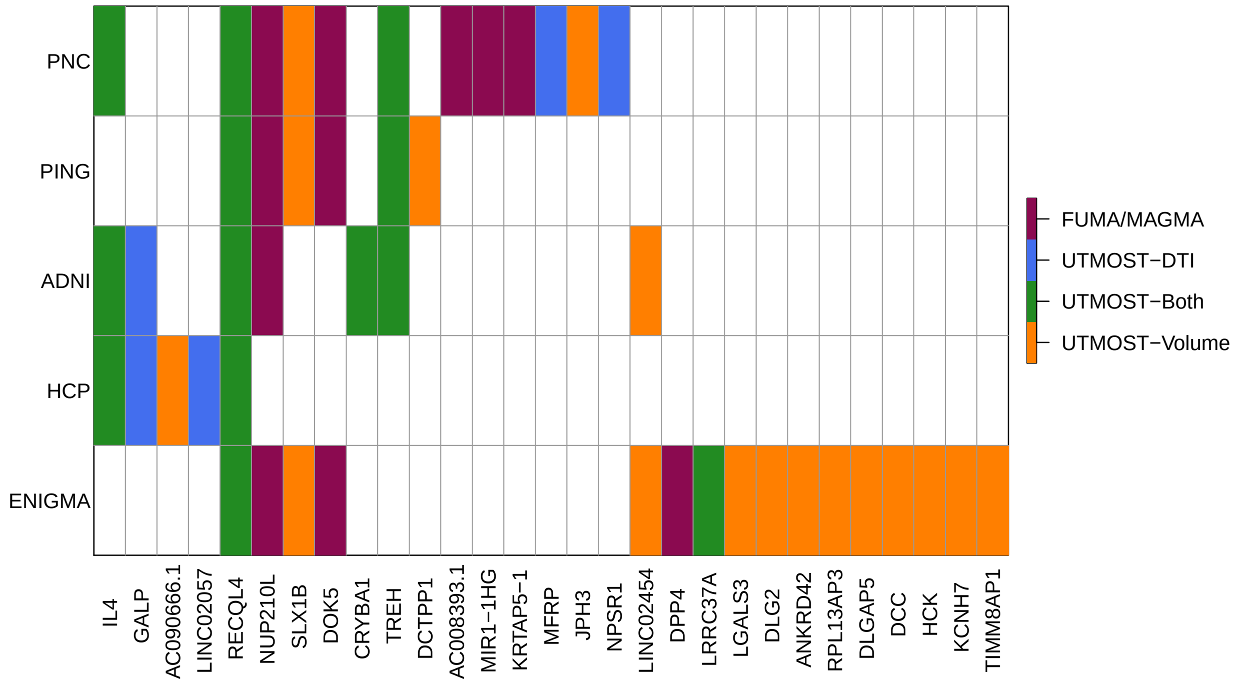


**Supplementary Figure 4: UKB significant genes that can be validated in one or more of the five validation datasets (n=537 subjects for PNC, 860 subjects for ADNI, 461 subjects for PING, 334 subjects for HCP, and 13,193 subjects for ENIGMA).** FUMA/MAGMA: genes identified in FUMA or MAGMA analysis; UTMOST-DTI: genes identified in cross-tissue TWAS analysis for DTI parameters; UTMOST-Volume: genes identified in cross-tissue TWAS analysis for ROI volumes; UTMOST-Both: genes identified in cross-tissue TWAS analysis for both DTI parameters and ROI volumes.

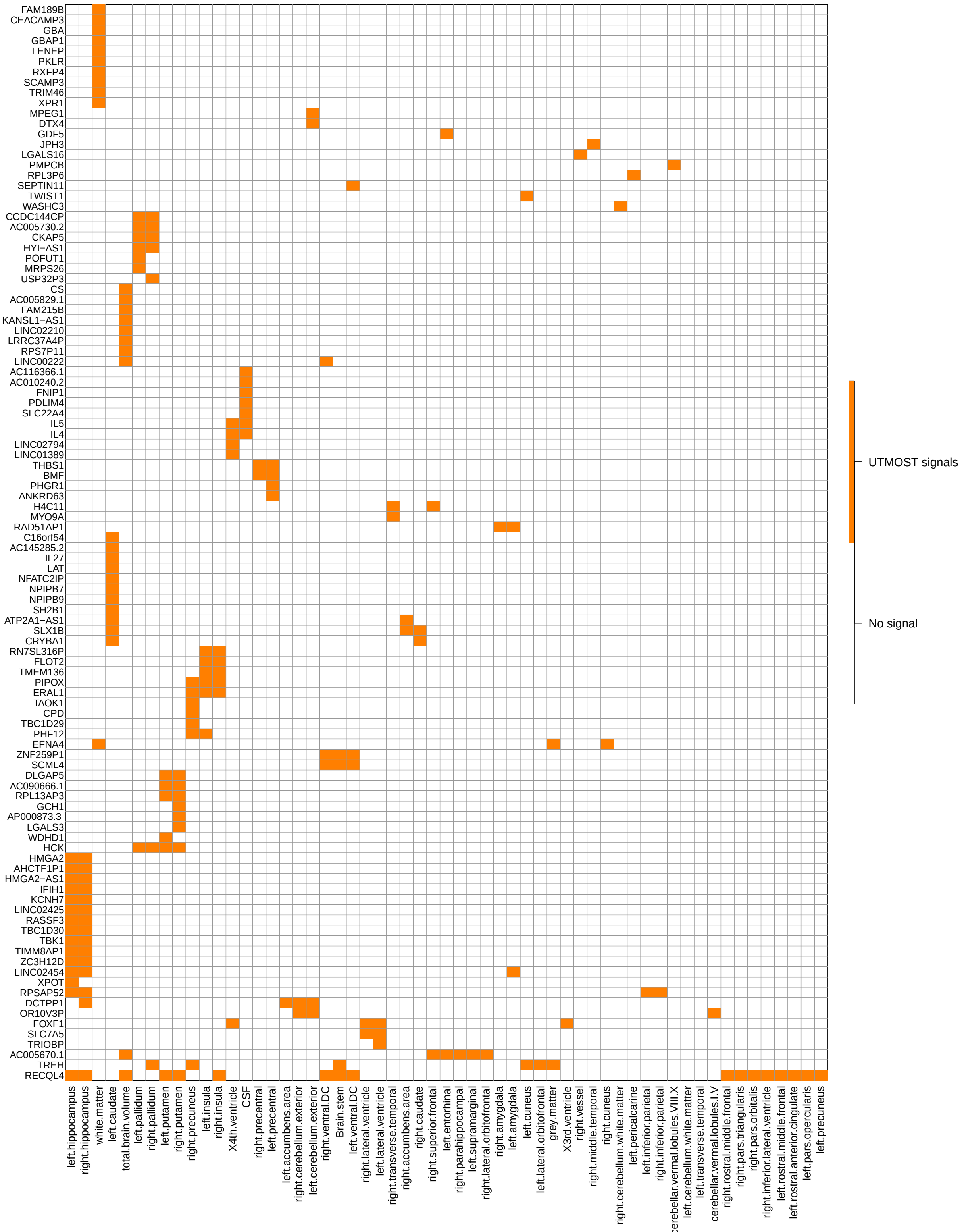


**Supplementary Figure 5: Additional significant genes and their associations identified in UKB cross-tissue TWAS analysis of ROI volumes (n=19,629 subjects).** These genes were not identified in previous GWAS MAGMA and FUMA analysis of the same dataset.

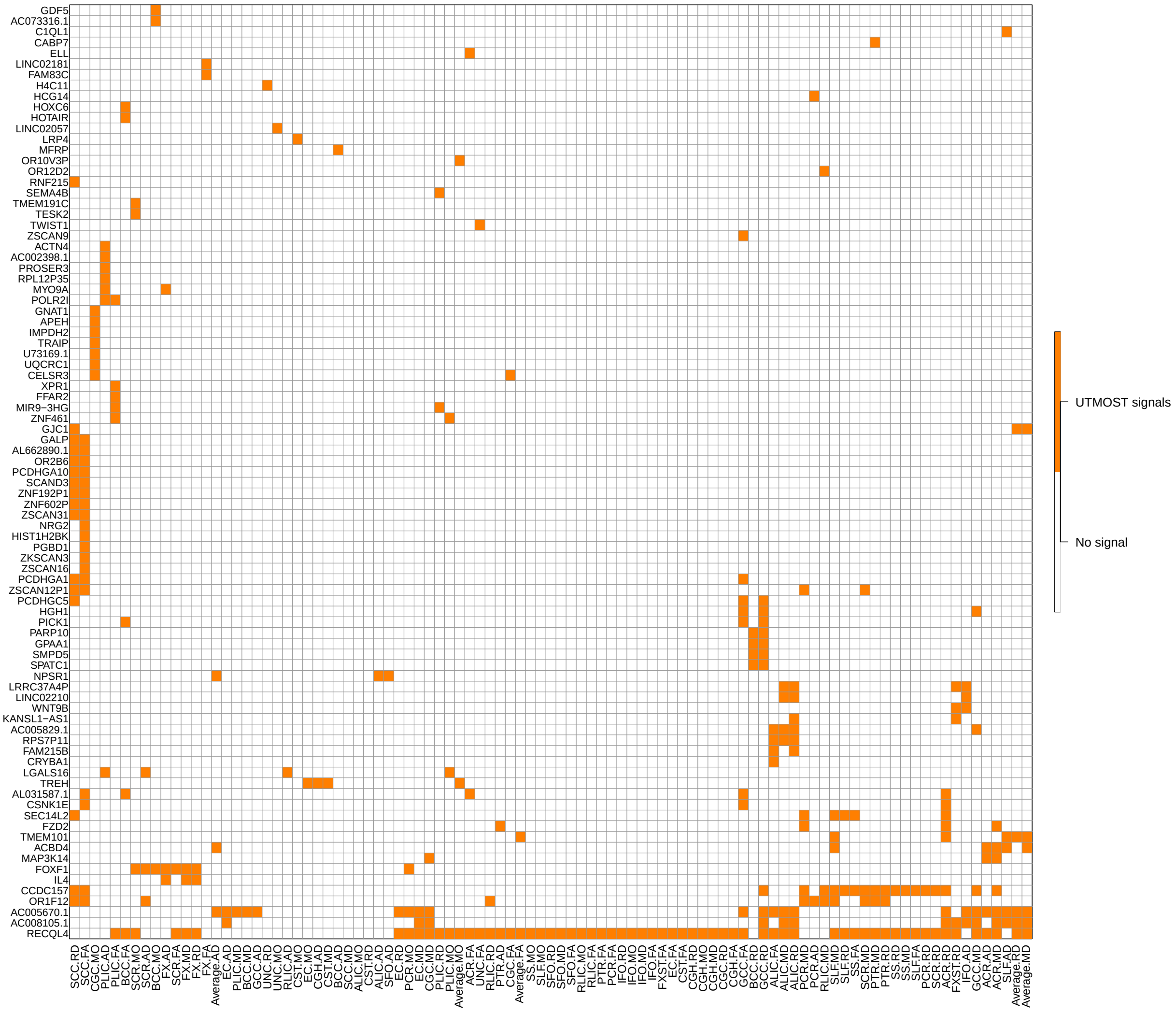


**Supplementary Figure 6: Additional significant genes and their associations identified in UKB cross-tissue TWAS analysis of ROI volumes (n=17,706 subjects).** These genes were not identified in previous GWAS MAGMA and FUMA analysis of the same dataset.

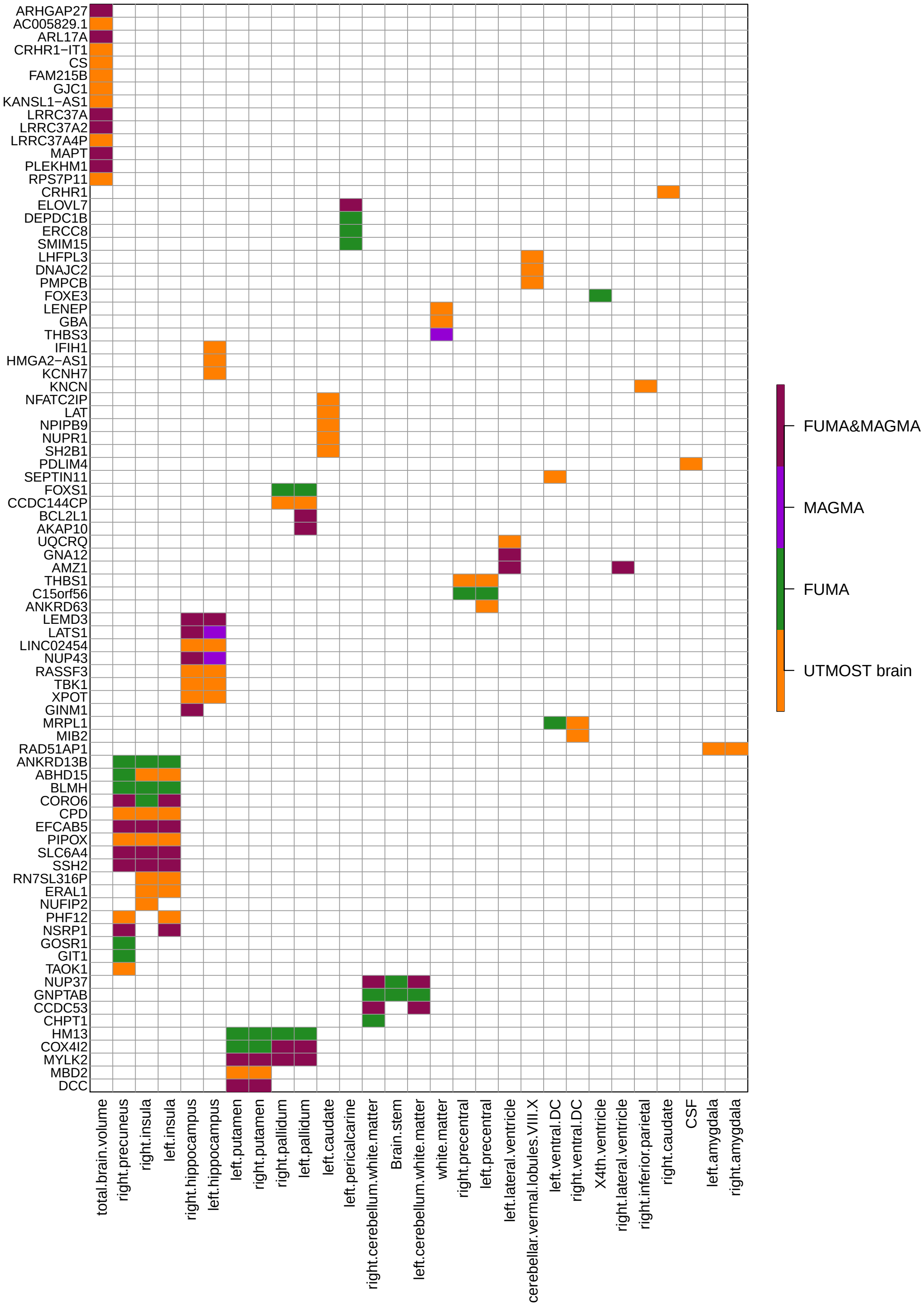


**Supplementary Figure 7: Significant gene-trait associations discovered in UKB brain tissue-specific TWAS analysis of ROI volumes (n=19,629 subjects).** FUMA: associations identified in FUMA; MAGMA: associations identified in MAGMA; FUMA&MAGMA: associations identified in both FUMA and MAGMA analysis; UTMOST brain: novel associations identified in brain tissue-specific TWAS analysis.

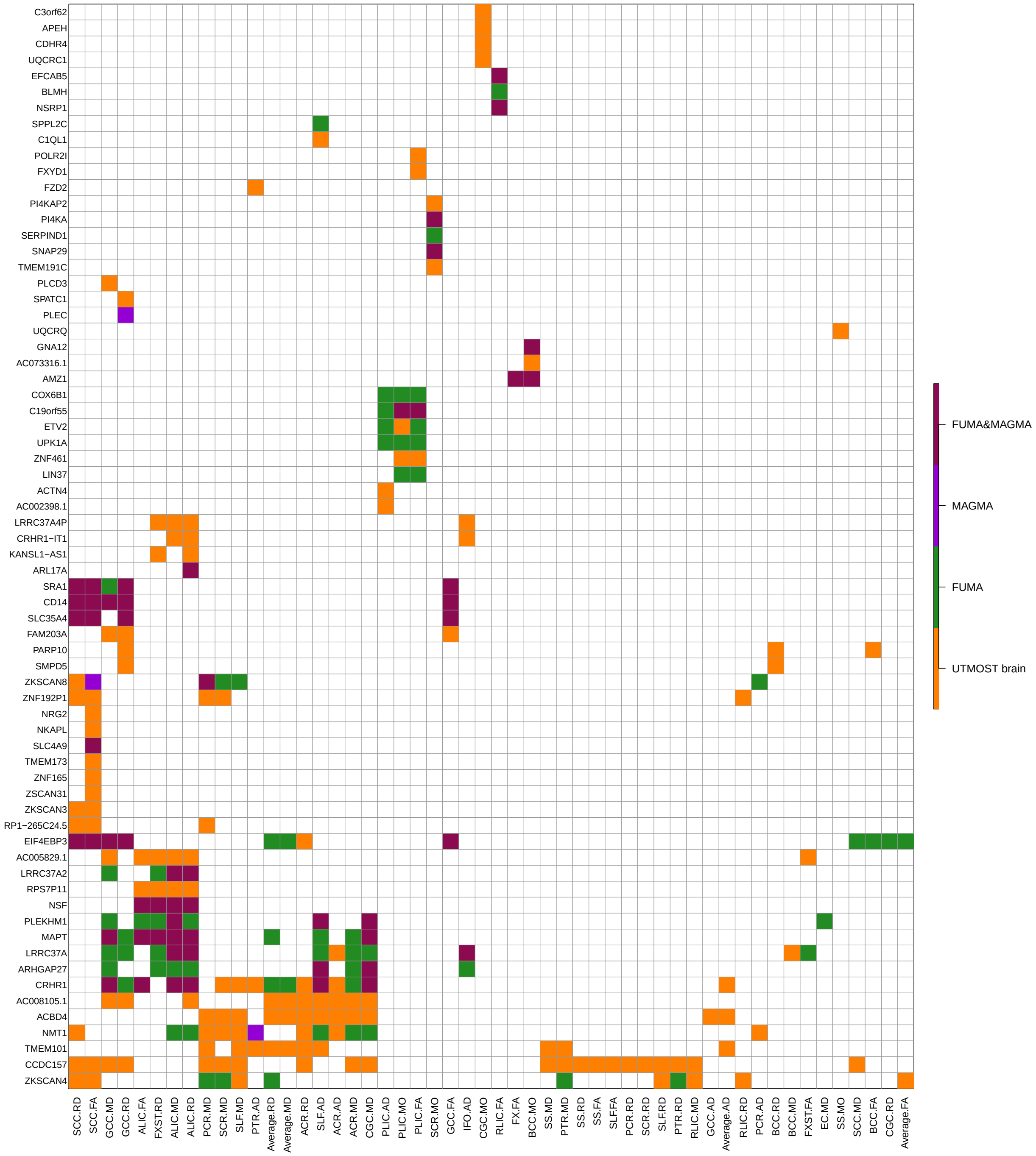


**Supplementary Figure 8: Significant gene-trait associations discovered in UKB brain tissue-specific TWAS analysis of DTI parameters (n=17,706 subjects).** FUMA: associations identified in FUMA; MAGMA: associations identified in MAGMA; FUMA&MAGMA: associations identified in both FUMA and MAGMA analysis; UTMOST brain: novel associations identified in brain tissue-specific TWAS analysis.

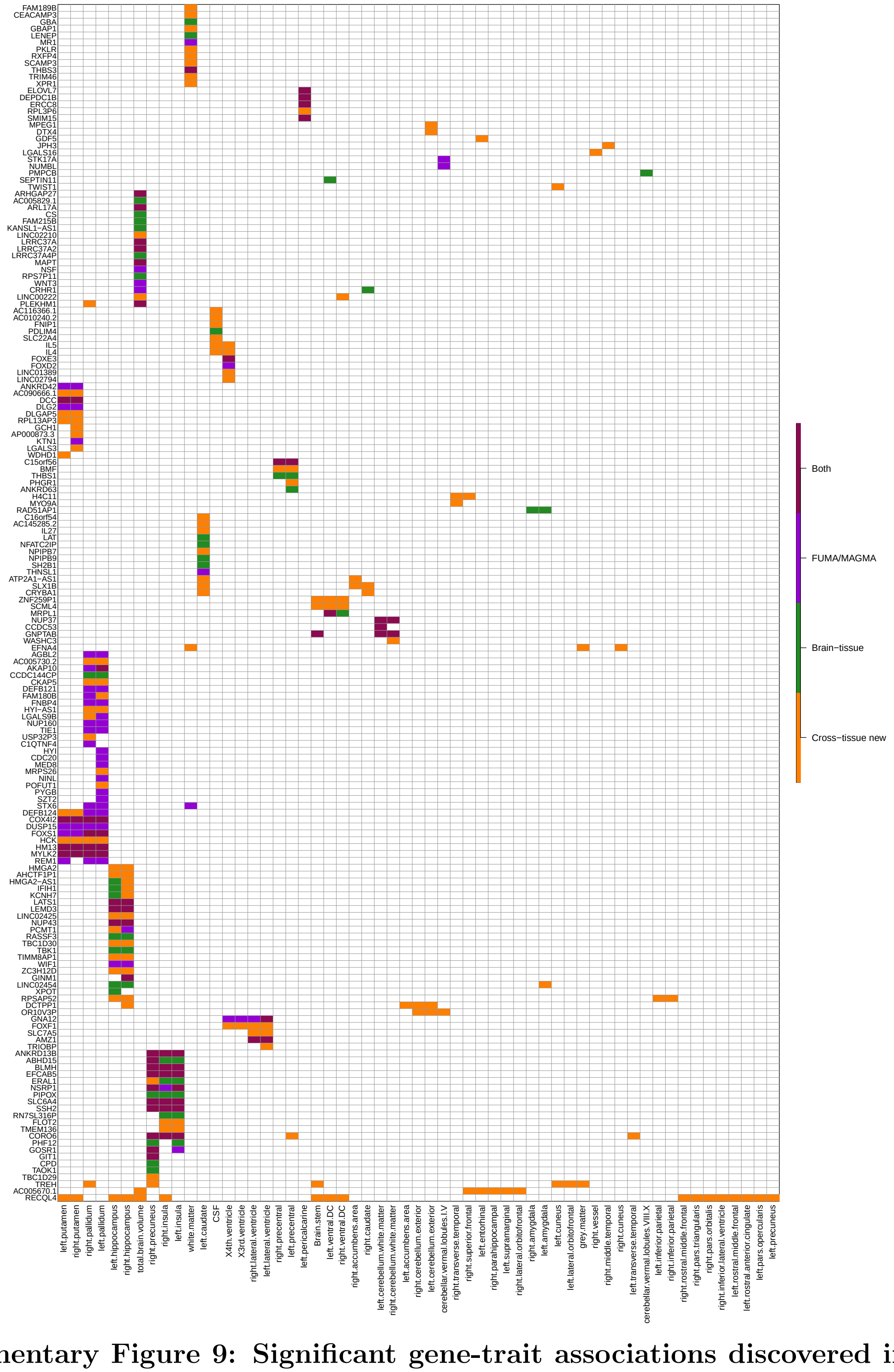


**Supplementary Figure 9: Significant gene-trait associations discovered in UKB cross-tissue TWAS analysis of ROI volumes (n=19,629 subjects).** Brain-tissue: associations identified in UKB brain tissue-specific analysis; Both: associations identified in both UKB brain tissue-specific analysis and FUMA or MAGMA analysis; Cross-tissue new: novel associations identifed in cross-tissue TWAS analysis.

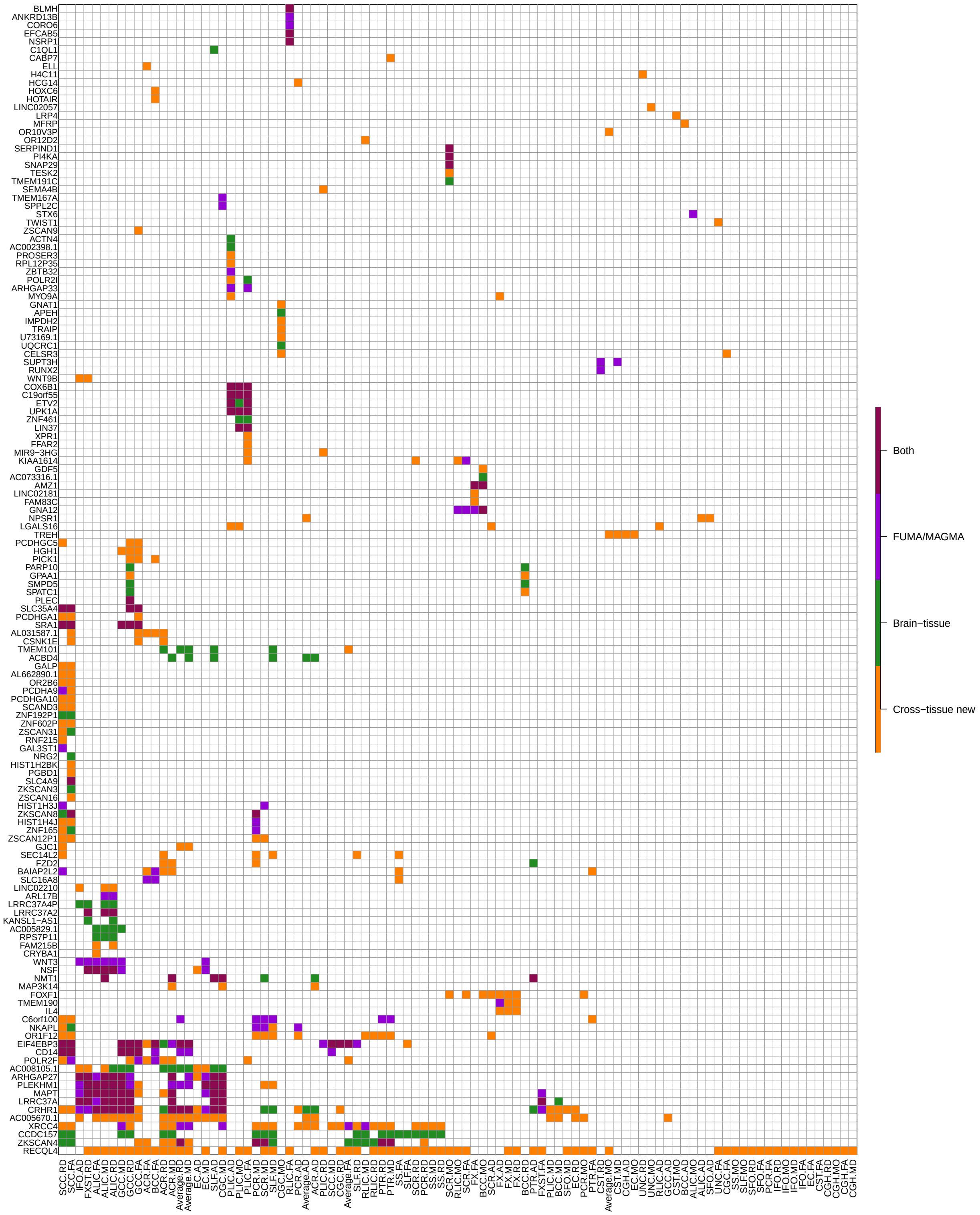


**Supplementary Figure 10: Significant gene-trait associations discovered in UKB cross-tissue TWAS analysis of DTI parameters (n=17,706 subjects).** Brain-tissue: associations identified in UKB brain tissue-specific analysis; Both: associations identified in both UKB brain tissue-specific analysis and FUMA or MAGMA analysis; Cross-tissue new: novel associations identifed in cross-tissue TWAS analysis.

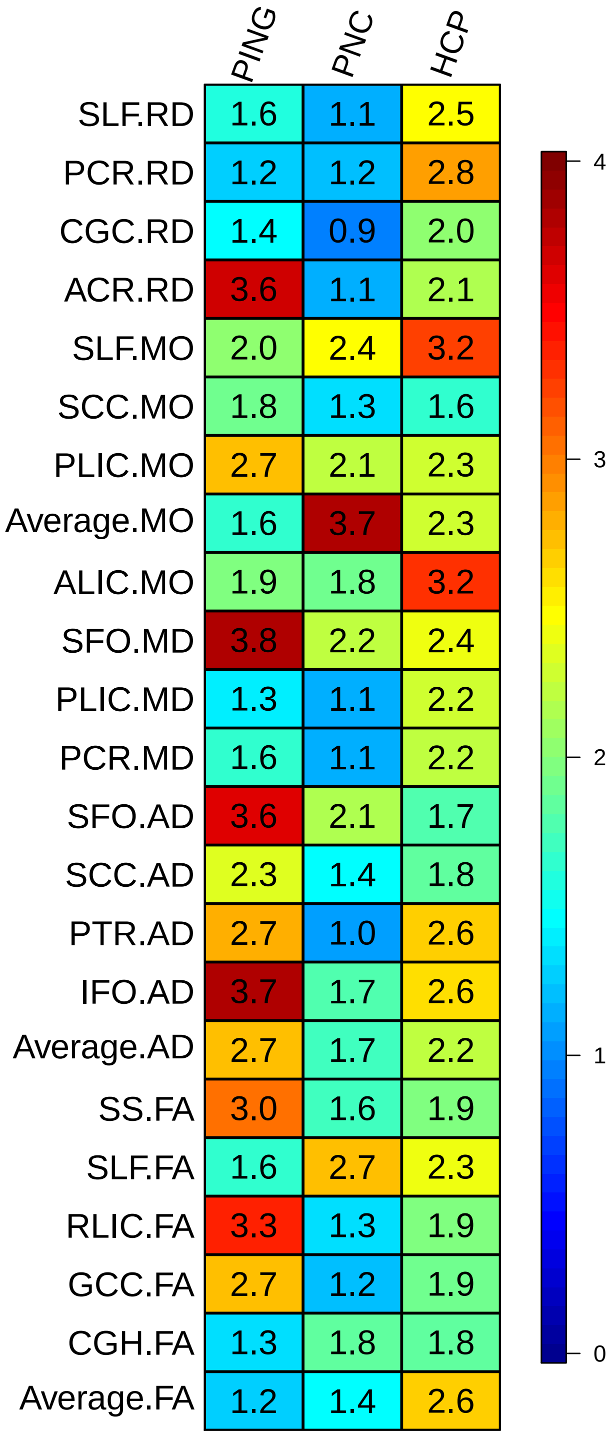


**Supplementary Figure 11: Prediction accuracy (incremental R-squared) of significant gene-based polygenic risk scores constructed by UKB-derived TWAS summary statistics (n=17,706 subjects) on the three independent datasets (PING, PNC, HCP).** We display the 23 DTI parameters that are significant in all the three datasets after the Bonferroni correction.

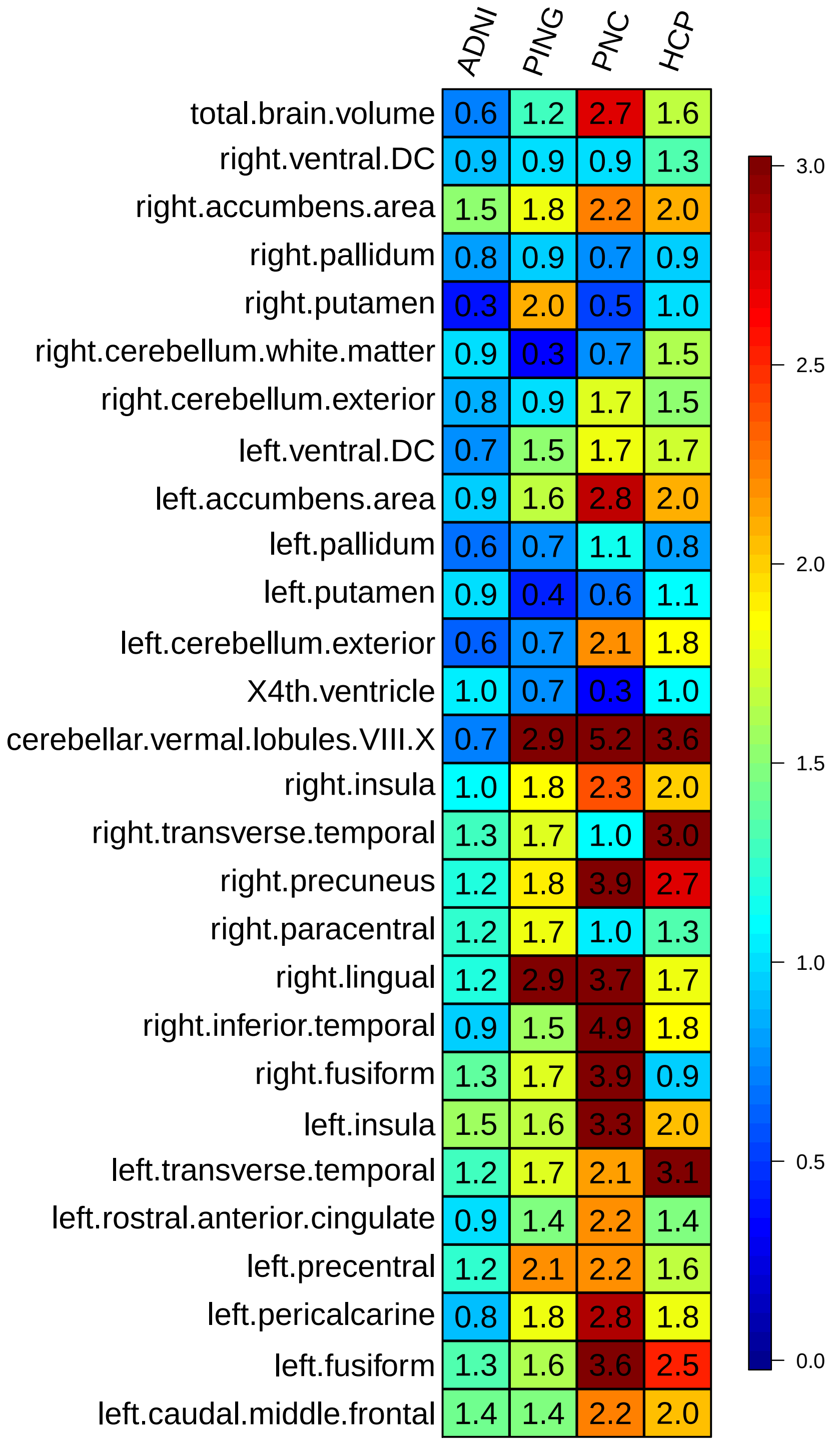


**Supplementary Figure 12: Prediction accuracy (incremental R-squared) of gene-based polygenic risk scores constructed by UKB-derived TWAS summary statistics conditioning on variant-based PRS constructed by UKB-derived GWAS summary statistics (n=19,629 subjects) on the four independent datasets (ADNI, PING, PNC, HCP).** We display the 28 ROI volumes whose TWAS PRS are significant in all the four datasets after the Bonferroni correction.

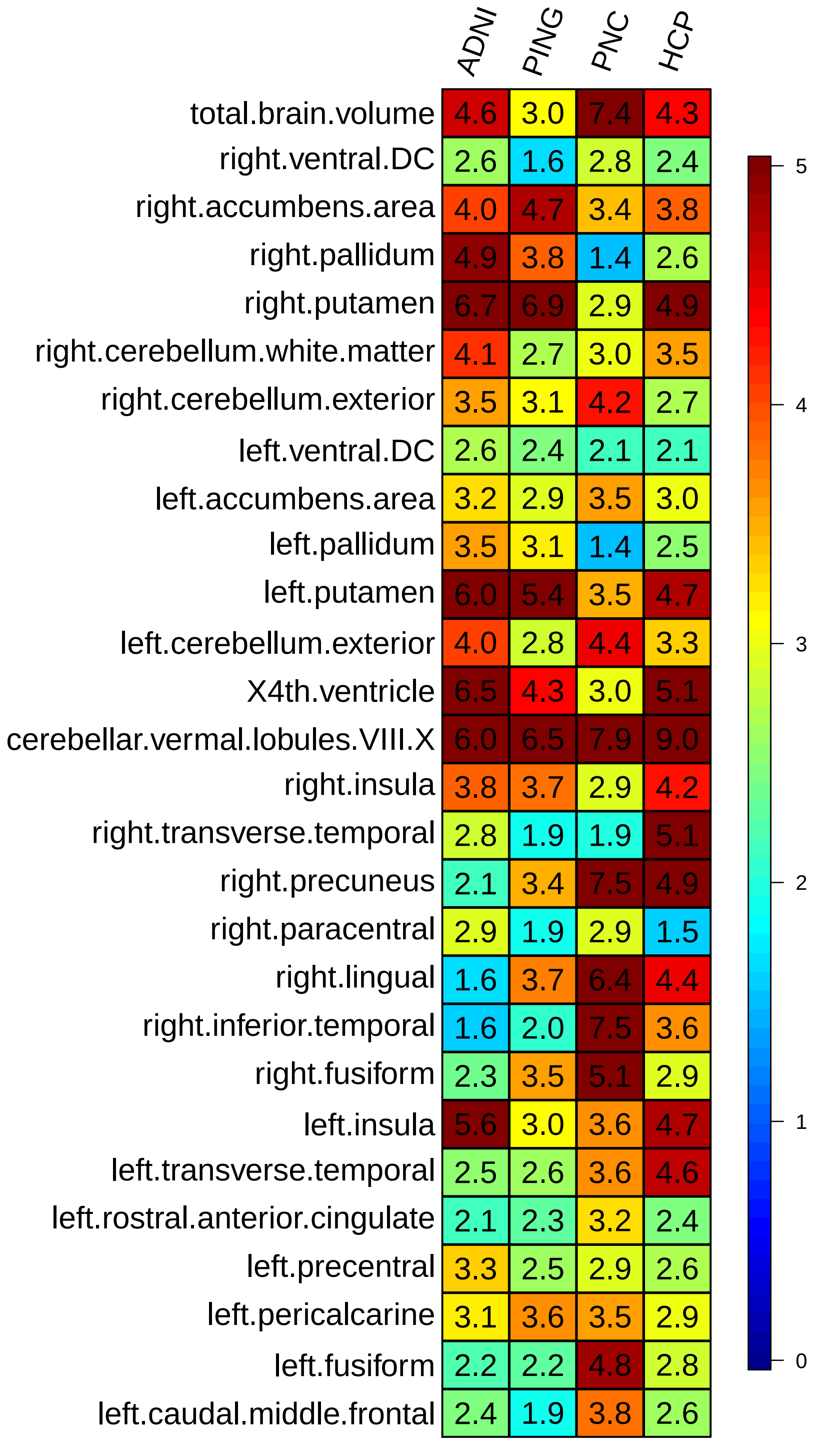


**Supplementary Figure 13: Prediction accuracy (incremental R-squared) of both gene-based polygenic risk scores constructed by UKB-derived TWAS summary statistics and variant-based PRS constructed by UKB-derived GWAS summary statistics (n=19,629 subjects) on the four independent datasets (ADNI, PING, PNC, HCP).** We display the 28 ROI volumes whose TWAS PRS are significant in all the four datasets after the Bonferroni correction.

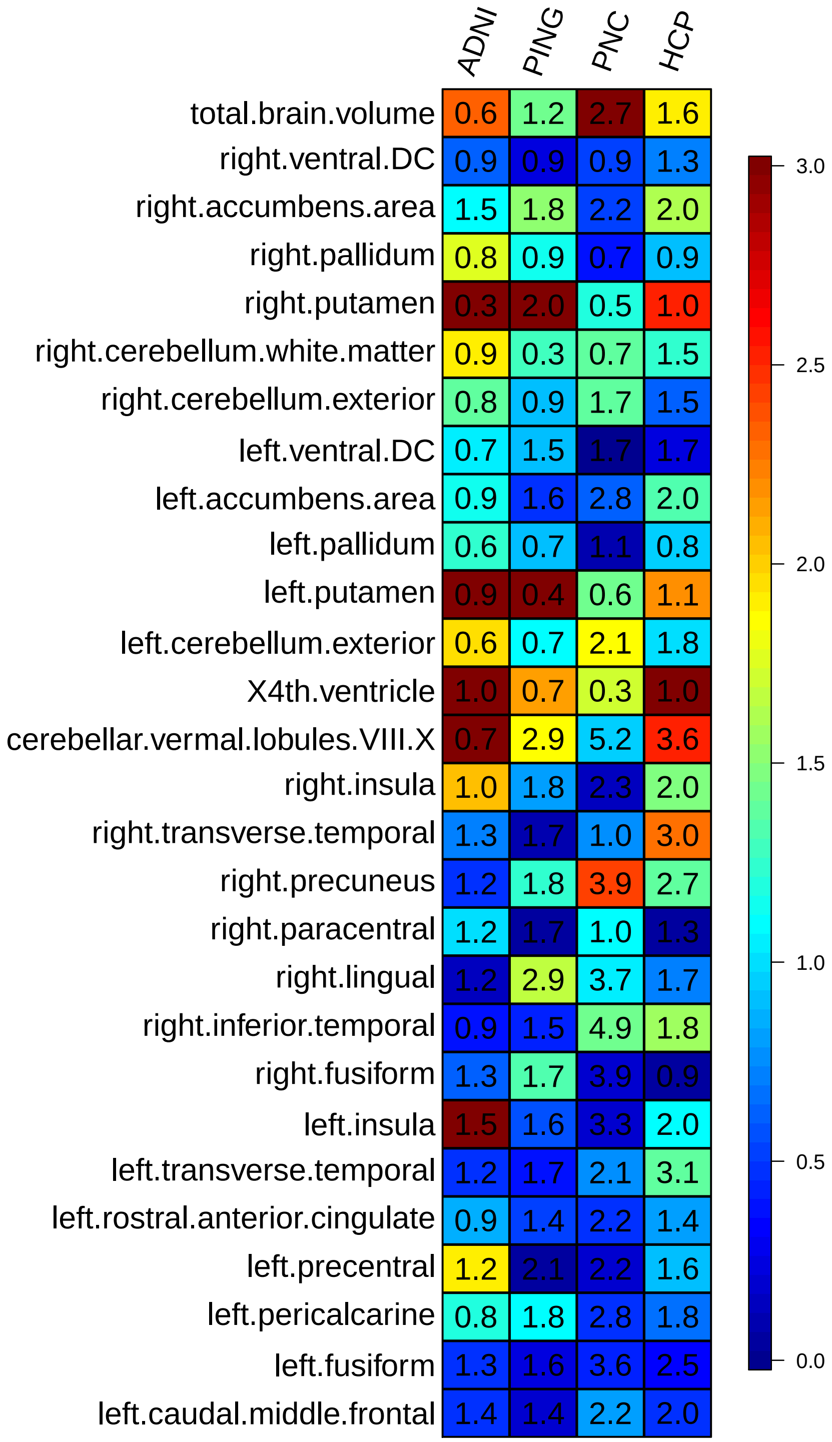


**Supplementary Figure 14: Prediction accuracy (incremental R-squared) of variant-based PRS constructed by UKB-derived GWAS summary statistics conditioning on gene-based polygenic risk scores constructed by UKB-derived TWAS summary statistics (n=19,629 subjects) on the four independent datasets (ADNI, PING, PNC, HCP).** We display the 28 ROI volumes whose TWAS PRS are significant in all the four datasets after the Bonferroni correction.

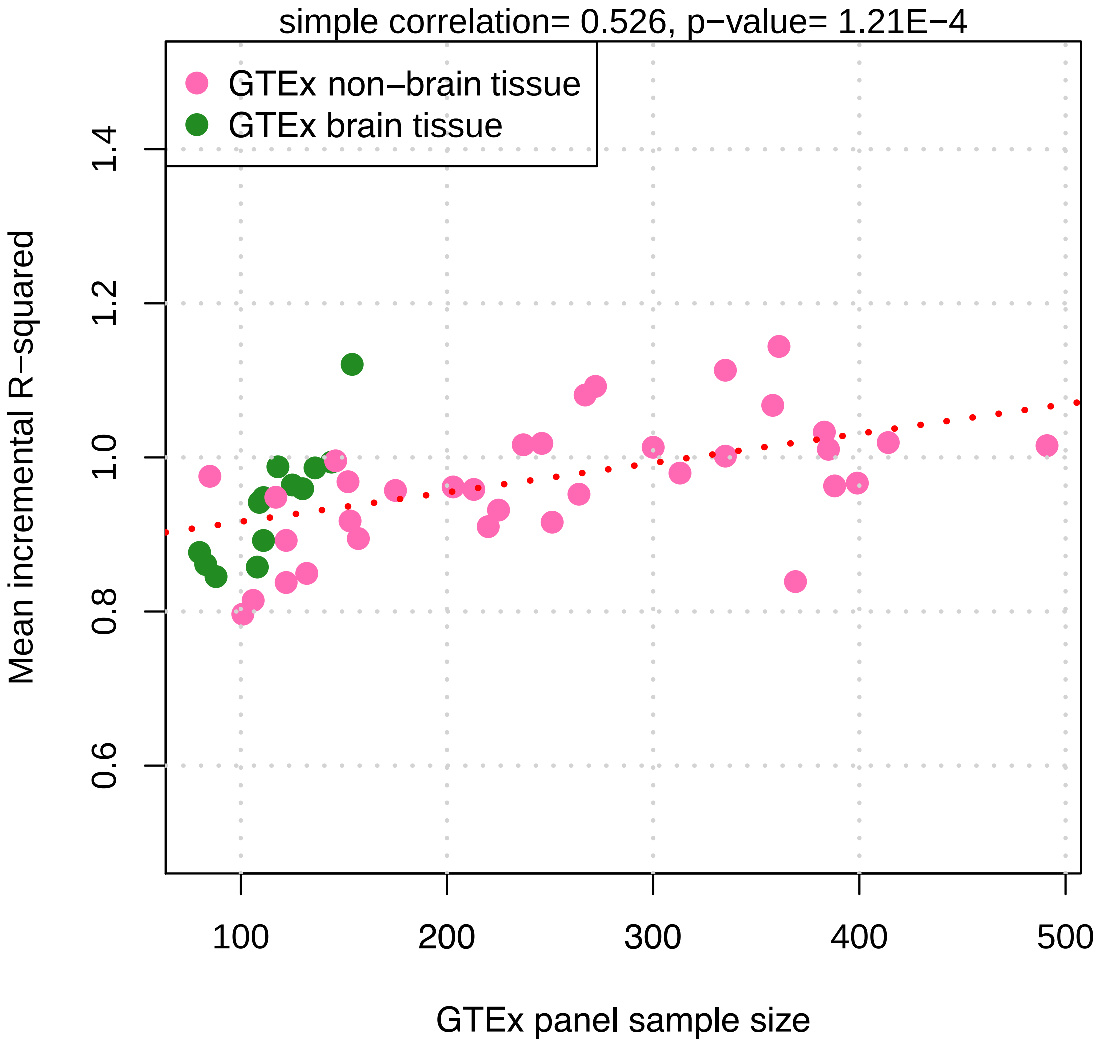


**Supplementary Figure 15: Relationship between mean incremental prediction R-squared of significant gene-based polygenic risk scores and the sample size of reference panels.** The polygenic risk scores were constructed on each GTEx reference panel by UKB-derived GWAS summary statistics (n=19,629 subjects for ROI volumes and 17,706 for DTI parameters, respectively)

# 2 Supplementary Note

## 2.1 Detailed steps to construct and evaluate Gene-based PRS

### 2.1.1 Obtain imputed gene expression

The FUSION [4] software was used to impute gene expression levels in UKB, ADNI, HCP, PNC, and PING datasets using individual-level genetic data. Specifically, we downloaded the pre-computed expression reference weights from `http://gusevlab.org/projects/fusion/` for 52 non-TCGA reference panels (13 GETx v7 brain tissues, 35 GTEx v7 other tissues, 1 non-GETx brain tissue, and 3 non-GETx other tissues). Then, we generated individual-level imputed gene expression for each reference panel using the *utilsmake_score.R* script from FUSION and PLINK.

### 2.1.2 Estimate the effect size of imputed gene expression

We used the UKB dataset as our training data to estimate the effect size of each imputed gene expression for each of the 52 reference panel. The effect size was estimated in a linear regression model for each gene, while adjusting for the age (at imaging), age-squared, sex, age-sex interaction, age-squared-sex interaction, as well as the top 40 genetic principle components. For ROI volumes, we also included total brain volume (for ROIs other than total brain volume itself) as a covariate. We also saved the associated p-value for each estimated effect size, which was used to threshold genes in next step.

### 2.1.3 Construct the gene-based PRS

With effect sizes estimated from the UKB dataset, we generated the gene-based TWAS PRS in ADNI, HCP, PNC, and PING datasets by summarizing across imputed gene expressions, weighted by their effect sizes. Specifically, the gene-based TWAS PRS for subject $i$, reference panel $j$, and given p-value threshold $c_k$ can be expressed as

$$PRS_{ijk} = \sum_l \omega_{jl} \cdot G_{ijl} \cdot \mathbf{1}(Pval_{jl} < c_k),$$

where $\omega_{jl}$ is the estimated effect size of gene $l$ in reference panel $j$, $Pval_{jl}$ is the associated p-value of $\omega_{jl}$, $G_{ijl}$ is the imputed gene expression of gene $l$ in reference panel $j$ for subject $i$, and $c_k$ is the $k$th p-value threshold. We tried 17 p-value thresholds for gene selection: 1, 0.8, 0.5, 0.4, 0.3, 0.2, 0.1, 0.08, 0.05, 0.02, 0.01, 0.001, $10^{-4}$, $10^{-5}$, $10^{-6}$, $10^{-7}$, and $5 \times 10^{-8}$.

### 2.1.4 Evaluate the gene-based PRS

The prediction accuracy of using gene-based PRS to predict neuroimaging trait was estimated and tested in linear regression model (R version 3.5.0), adjusting for the effects of age and

sex. Specifically, we constructed the following two linear models

$$Phenotype \sim covariates\ (m1),$$

and

$$Phenotype \sim TWAS\_PRS + covariates\ (m2),$$

and the difference of the R-squared between $m2$ and $m1$ was regarded as the phenotypic variation that can be additionally explained by gene-based PRS. We reported the best prediction performance across the 17 p-value thresholds. Next, we considered the following 2 linear models

$$Phenotype \sim GWAS\_PRS + covariates\ (m3),$$

and

$$Phenotype \sim TWAS\_PRS + GWAS\_PRS + covariates\ (m4).$$

We measured the additional prediction accuracy of TWAS PRS conditioning on GWAS PRS by comparing the R-squared of models $m4$ and $m3$, the additional prediction accuracy of GWAS PRS conditioning on TWAS PRS using models $m4$ and $m2$, and calculated the additional phenotypic variation that can be jointly explained by GWAS and TWAS PRS using models $m4$ and $m1$.

### 2.1.5 Gene-Based PRS with colocalization

We have also incorporated colocalization information into the gene-based PRS and checked the robustness of the performance. The colocalization information was estimated by MOLOC analysis [3]. Specifically, we input the UKB GWAS summary statistics and meta-analyzed non-UKB GWAS summary statistics, and then performed MOLOC using each of the 49 GTEx v8 tissues separately for all of our 211 neuroimaging traits. For each gene, MOLOC estimated the posterior probability (PP) of having a colocalized signal shared among the three datasets (UKB GWAS, non-UKB GWAS, and GTEx eQTL). We used this PP to further weight the genes when constructing the gene-based PRS. That is, we let MOLOC-weighted gene-based PRS to be $\sum_i \omega_i \widehat{\beta}_i \widehat{\mathrm{G}}$, where $\omega_i$ is the maximum of PP across different reference panels for the $i$th gene, $\widehat{\beta}_i$ is the estimated gene effect size of the $i$th gene from the training data (UKB GWAS), and $\widehat{\mathrm{G}}$ is the imputed gene expression of the ith gene in the testing data (non-UKB GWAS). The only difference between MOLOC-weighted PRS and our original gene-level PRS is that our original gene-level PRS did not use $\omega_i$s. For genes not present in the MOLOC results, we set $\omega_i$ to be $0.5 \times$ minimum PP across all the genes. Intuitively, $\omega_i$ uses the colocalization information to weight these genes and prioritizes the genes with high PP. The mean R-squared of MOLOC-weighted PRS for our neuroimaging traits was 2.27%, which was similar to the mean R-squared of our original gene-level PRS (2.34%, Wilcoxon rank test p-value = 0.88). Overall, our results may suggest that our

gene-based PRS analysis is resilient to typical confounding factors or distinct causal variants related by LD.

## 2.2 GWAS on validation cohorts

In this study, we made use of summary-level data from six studies for validation, whose GWAS analysis details can be found in [9, 10] for HCP, PING, PNC, and ADNI; and [1, 5] for ENIGMA (ENIGMA2 and the ENIGMA-CHARGE collaboration).

The GWAS of HCP, PING, PNC, and ADNI cohorts only considered the unrelated European ancestry individuals (based on self-reported race, ethnic, and family information) in GWAS [9, 10]. The following genetic variants data quality controls (QCs) were performed on each dataset: 1) exclude subjects with more than 10% missing genotypes; 2) exclude variants with minor allele frequency less than 0.01; 3) exclude variants with larger than 10% missing genotyping rate; 4) exclude variants that failed the Hardy-Weinberg test at $1 \times 10^{-7}$ level; and 5) remove variants with imputation INFO score less than 0.8.

ENIGMA brought together numerous studies and performed meta-analysis GWAS [1, 5]. The main discovery GWAS in ENIGMA was performed on European ancestry. Studies of unrelated individuals performed a linear regression analyses whereas studies of related individuals used linear mixed models to account for familial relationships. In ENIGMA, both samples as well as variants underwent similar quality control procedures based on genetic homogeneity, call rate (less than 95%), minor allele frequency (MAF $< 0.01$), and Hardy-Weinberg Equilibrium (HWE p-value less than $1 \times 10^{-6}$). Good quality variants were used as input for imputation to the 1000 Genomes reference panel (phase 1, version 3). Variants that were poorly imputed ($R^2 < 0.5$) or uncommon (MAF $< 0.5\%$) were removed.

## 2.3 UTMOST training using GTEx v8 data

The cross-tissue gene expression imputation models were trained based on genotype data, expression data, and other covariates downloaded from GTEx portal (GTEx V8) [2]. The genotype data were pruned with PLINK to remove highly correlated SNPs [7]. The expression levels for each sample were adjusted for other covariates including sex, sequencing platform, top three principal components of genotype data, and the top probabilistic estimation of expression residuals (PEER) to remove potential confounding effects [8]. The models were then trained using CTIMP (Cross Tissue gene expression IMPutation) across 49 tissues [6].

In terms of the cis-SNPs used in imputation models, we used rsid as the SNP reference. For a given tissue, the imputation models for a specific gene were saved as multiple records in the database. Each record corresponded to the weight of an rsid with respect to a gene. As for the range of cis-SNPs for a gene, we used the corresponding gene reference build (GRCh38 for GTEx v8) to identify the cis-SNPs.

## PING Methods

Part of the data used in the preparation of this article were obtained from the Pediatric Imaging, Neurocognition and Genetics (PING) Study database (http://ping.chd.ucsd.edu/). PING was launched in 2009 by the National Institute on Drug Abuse (NIDA) and the Eunice Kennedy Shriver National Institute Of Child Health & Human Development (NICHD) as a 2-year project of the American Recovery and Reinvestment Act. The primary goal of PING has been to create a data resource of highly standardized and carefully curated magnetic resonance imaging (MRI) data, comprehensive genotyping data, and developmental and neuropsychological assessments for a large cohort of developing children aged 3 to 20 years. The scientific aim of the project is, by openly sharing these data, to amplify the power and productivity of investigations of healthy and disordered development in children, and to increase understanding of the origins of variation in neurobehavioral phenotypes. For up-to-date information, see http://ping.chd.ucsd.edu/.

## ADNI Methods

Data used in the preparation of this article were obtained from the Alzheimer's Disease Neuroimaging Initiative (ADNI) database (http://adni.loni.usc.edu). The ADNI was launched in 2003 by the National Institute on Aging (NIA), the National Institute of Biomedical Imaging and Bioengineering (NIBIB), the Food and Drug Administration (FDA), private pharmaceutical companies and non-profit organizations, as a 60 million, 5-year public-private partnership. The primary goal of ADNI has been to test whether serial magnetic resonance imaging (MRI), positron emission tomography (PET), other biological markers, and clinical and neuropsychological assessment can be combined to measure the progression of mild cognitive impairment (MCI) and early Alzheimer's disease (AD). Determination of sensitive and specific markers of very early AD progression is intended to aid researchers and clinicians to develop new treatments and monitor their effectiveness, as well as lessen the time and cost of clinical trials.

The Principal Investigator of this initiative is Michael W. Weiner, MD, VA Medical Center and University of California – San Francisco. ADNI is the result of efforts of many co-investigators from a broad range of academic institutions and private corporations, and subjects have been recruited from over 50 sites across the U.S. and Canada. The initial goal of ADNI was to recruit 800 subjects but ADNI has been followed by ADNI-GO and ADNI-2. To date these three protocols have recruited over 1500 adults, ages 55 to 90, to participate in the research, consisting of cognitively normal older individuals, people with early or late MCI, and people with early AD. The follow up duration of each group is specified in the protocols for ADNI-1, ADNI-2 and ADNI-GO. Subjects originally recruited for ADNI-1 and ADNI-GO had the option to be followed in ADNI-2. For up-to-date information, see www.adni-info.org.

# Pediatric Imaging, Neurocognition and Genetics (PING) Authors

Connor McCabe[1], Linda Chang[2], Natacha Akshoomoff[3], Erik Newman[1], Thomas Ernst[2], Peter Van Zijl[4], Joshua Kuperman[5], Sarah Murray[6], Cinnamon Bloss[6], Mark Appelbaum[1], Anthony Gamst[1], Wesley Thompson[3], Hauke Bartsch[5].

# Alzheimer's Disease Neuroimaging Initiative (ADNI) Authors

Michael Weiner[7], Paul Aisen[1], Ronald Petersen[8], Clifford R. Jack Jr[8], William Jagust[9], John Q. Trojanowki[10], Arthur W. Toga[11], Laurel Beckett[12], Robert C. Green[13], Andrew J. Saykin[14], John Morris[15], Leslie M. Shaw[10], Zaven Khachaturian[16], Greg Sorensen[17], Maria Carrillo[18], Lew Kuller[19], Marc Raichle[15], Steven Paul[20], Peter Davies[21], Howard Fillit[22], Franz Hefti[23], Davie Holtzman[15], M. Marcel Mesulman[24], William Potter[25], Peter J. Snyder[26], Adam Schwartz[27], Tom Montine[28], Ronald G. Thomas[1], Michael Donohue[1], Sarah Walter[1], Devon Gessert[1], Tamie Sather[1], Gus Jiminez[1], Danielle Harvey[12], Matthew Bernstein[8], Nick Fox[29], Paul Thompson[11], Norbert Schuff[7], Charles DeCarli[12], Bret Borowski[8], Jeff Gunter[8], Matt Senjem[8], Prashanthi Vemuri[8], David Jones[8], Kejal Kantarci[8], Chad Ward[8], Robert A. Koeppe[30], Norm Foster[31], Eric M. Reiman[32], Kewei Chen[32], Chet Mathis[19], Susan Landau[9], Nigel J. Cairns[15], Erin Householder[15], Lisa Taylor-Reinwald[15], Virginia M.Y. Lee[10], Magdalena Korecka[10], Michal Figurski[10], Karen Crawford[11], Scott Neu[11], Tatiana M. Foroud[14], Steven Potkin[33], Li Shen[14], Kelley Faber[14], Sungeun Kim[14], Kwangsik Nho[14], Leon Thal[1], Richard Frank[34], Neil Buckholtz[35], Marilyn Albert[36], John Hsiao[35].

[1]UC San Diego, La Jolla, CA 92093, USA. [2]U Hawaii, Honolulu, HI 96822, USA. [3]Department of Psychiatry, University of California, San Diego, La Jolla, California 92093, USA. [4]Kennedy Krieger Institute, Baltimore, MD 21205, USA. [5]Multimodal Imaging Laboratory, Department of Radiology, University of California San Diego, La Jolla, California 92037, USA. [6]Scripps Translational Science Institute, La Jolla, CA 92037, USA. [7]UC San Francisco, San Francisco, CA 94143, USA. [8]Mayo Clinic, Rochester, MN 55905, USA. [9]UC Berkeley, Berkeley, CA 94720-5800, USA. [10]U Pennsylvania, Philadelphia, PA 19104, USA. [11]USC, University of Southern California, Los Angeles, CA 90033, USA. [12]UC Davis, Davis, CA 95616, USA. [13]Brigham and Women s Hospital/Harvard Medical School, Boston MA 02115, USA. [14]Indiana University, Indianapolis, IN 46202-5143, USA. [15]Washington University St. Louis, St. Louis, MO 63130, USA. [16]Prevent Alzheimer's Disease 2020, Rockville, MD 20850, USA. [17]Siemens [18]Alzheimer's Association, Chicago, IL 60601,

USA. [19]University of Pittsburgh, Pittsburgh, PA 15260, USA. [20]Cornell University, Ithaca, NY 14850, USA. [21]Albert Einstein College of Medicine of Yeshiva University, Bronx, NY 10461, USA. [22]AD Drug Discovery Foundation, New York, NY 10019, USA. [23]Acumen Pharmaceuticals, Livermore, California 94551, USA. [24]Northwestern University, Evanston, IL 60208, USA. [25]National Institute of Mental Health, Bethesda, MD 20892-9663, USA. [26]Brown University, Providence, RI 02912, USA. [27]Eli Lilly, Indianapolis, Indiana 46285, USA. [28]University of Washington, Seattle, WA 98195, USA. [29]University of London, London WC1E 7HU, UK. [30]University of Michigan, Ann Arbor, MI 48109, USA. [31]University of Utah, Salt Lake City, UT 84112, USA. [32]Banner Alzheimer's Institute, Phoenix, AZ 85006, USA. [33]UC Irvine, Irvine, CA 92697, USA. [34]General Electric [35]National Institute on Aging/National Institutes of Health, Bethesda, MD 20892, USA. [36]The Johns Hopkins University, Baltimore, MD 21218, USA.

# References

[1] H. H. Adams, D. P. Hibar, V. Chouraki, J. L. Stein, P. A. Nyquist, M. E. Rentería, S. Trompet, A. Arias-Vasquez, S. Seshadri, S. Desrivières, et al. Novel genetic loci underlying human intracranial volume identified through genome-wide association. *Nature neuroscience*, 19(12):1569, 2016.

[2] G. Consortium et al. The gtex consortium atlas of genetic regulatory effects across human tissues. *Science*, 369(6509):1318–1330, 2020.

[3] C. Giambartolomei, J. Zhenli Liu, W. Zhang, M. Hauberg, H. Shi, J. Boocock, J. Pickrell, A. E. Jaffe, C. Consortium, B. Pasaniuc, et al. A bayesian framework for multiple trait colocalization from summary association statistics. *Bioinformatics*, 34(15):2538–2545, 2018.

[4] A. Gusev, A. Ko, H. Shi, G. Bhatia, W. Chung, B. W. Penninx, R. Jansen, E. J. De Geus, D. I. Boomsma, F. A. Wright, et al. Integrative approaches for large-scale transcriptome-wide association studies. *Nature Genetics*, 48(3):245–252, 2016.

[5] D. P. Hibar, J. L. Stein, M. E. Renteria, A. Arias-Vasquez, S. Desrivières, N. Jahanshad, R. Toro, K. Wittfeld, L. Abramovic, M. Andersson, et al. Common genetic variants influence human subcortical brain structures. *Nature*, 520(7546):224, 2015.

[6] Y. Hu, Q. Lu, W. Liu, Y. Zhang, M. Li, and H. Zhao. Joint modeling of genetically correlated diseases and functional annotations increases accuracy of polygenic risk prediction. *PLoS genetics*, 13(6):e1006836, 2017.

[7] S. Purcell, B. Neale, K. Todd-Brown, L. Thomas, M. A. Ferreira, D. Bender, J. Maller, P. Sklar, P. I. De Bakker, M. J. Daly, et al. Plink: a tool set for whole-genome association

and population-based linkage analyses. *The American Journal of Human Genetics*, 81(3):559–575, 2007.

[8] O. Stegle, L. Parts, M. Piipari, J. Winn, and R. Durbin. Using probabilistic estimation of expression residuals (peer) to obtain increased power and interpretability of gene expression analyses. *Nature protocols*, 7(3):500, 2012.

[9] B. Zhao, T. Luo, T. Li, Y. Li, J. Zhang, Y. Shan, X. Wang, L. Yang, F. Zhou, Z. Zhu, et al. Genome-wide association analysis of 19,629 individuals identifies variants influencing regional brain volumes and refines their genetic co-architecture with cognitive and mental health traits. *Nature Genetics*, 51:1637–1644, 2019.

[10] B. Zhao, J. Zhang, J. G. Ibrahim, T. Luo, R. C. Santelli, Y. Li, T. Li, Y. Shan, Z. Zhu, F. Zhou, et al. Large-scale gwas reveals genetic architecture of brain white matter microstructure and genetic overlap with cognitive and mental health traits (n= 17,706). *Molecular psychiatry*, pages 1–13, 2019.
