# Supplementary material for: Transcriptome-wide association analysis of brain structures yields insights into pleiotropy with complex neuropsychiatric traits
Source: Nat Commun. 2021 May 17;12:2878. doi: 10.1038/s41467-021-23130-y (PMC8128893; doi:10.1038/s41467-021-23130-y)
Supplement: Supplementary file 4 — Description of Additional Supplementary Files [file 41467_2021_23130_MOESM4_ESM.pdf]

Description of Additional Supplementary Files

File Name: Supplementary Data 1
Description: List of significant gene-trait associations discovered in UKB cross-tissue TWAS analysis of 211 neuroimaging traits (n=19,629 subjects for ROI volumes and 17,706 for DTI parameters). The p-values are raw p-values of two-sided Generalized Berk-Jones (GBJ) test.

File Name: Supplementary Data 2
Description: List of significant gene-trait associations identified in ADNI cross-tissue TWAS analysis of 101 ROI volumes (n=860 subjects). The pvalues are raw p-values of two-sided Generalized Berk-Jones (GBJ) test.

File Name: Supplementary Data 3
Description: List of significant gene-trait associations identified in ENIGMA cross-tissue TWAS analysis of 8 ROI volumes (n=13,193 subjects). The p-values are raw p-values of two-sided Generalized Berk-Jones (GBJ) test.

File Name: Supplementary Data 4
Description: List of significant gene-trait associations discovered in HCP cross-tissue TWAS analysis of 211 neuroimaging traits (n=334 subjects for ROI volumes and 319 for DTI parameters). The p-values are raw p-values of two-sided Generalized Berk-Jones (GBJ) test

File Name: Supplementary Data 5
Description: List of significant gene-trait associations discovered in PING cross-tissue TWAS analysis of 211 neuroimaging traits (n=461 subjects for ROI volumes and 444 for DTI parameters). The p-values are raw p-values of two-sided Generalized Berk-Jones (GBJ) test.

File Name: Supplementary Data 6
Description: List of significant gene-trait associations discovered in PNC cross-tissue TWAS analysis of 211 neuroimaging traits (n=537 subjects for ROI volumes and 520 for DTI parameters). The p-values are raw p-values of two-sided Generalized Berk-Jones (GBJ) test.

File Name: Supplementary Data 7
Description: List of significant genes identified in UKB cross-tissue TWAS analysis of 211 neuroimaging traits (n=19,629 subjects for ROI volumes and 17,706 for DTI parameters) that were not discovered in previous GWAS of the same dataset.

File Name: Supplementary Data 8
Description: List of significant gene-trait associations discovered in UKB brain tissue-specific TWAS analysis of 211 neuroimaging traits (n=19,629 subjects for ROI volumes and 17,706 for DTI parameters). The pvalues are raw p-values of two-sided Generalized Berk-Jones (GBJ) test.

File Name: Supplementary Data 9
Description: The most significant GWAS variant-level signal corresponding to the gene-trait associations discovered in UKB cross-tissue TWAS analysis of 211 neuroimaging traits (n=19,629 subjects for ROI volumes and 17,706 for DTI parameters).

File Name: Supplementary Data 10

Description: The most significant GWAS variant-level signal corresponding to the gene-trait associations discovered in UKB brain tissuespecific TWAS analysis of 211 neuroimaging traits (n=19,629 subjects for ROI volumes and 17,706 for DTI parameters).

File Name: Supplementary Data 11
Description: Conditional analysis of significant gene-trait associations discovered in UKB cross-tissue TWAS analysis of 211 neuroimaging traits (n=19,629 subjects for ROI volumes and 17,706 for DTI parameters). The p-values are raw p-values of two-sided Generalized BerkJones (GBJ) test.

File Name: Supplementary Data 12
Description: Conditional analysis of significant gene-trait associations discovered in UKB brain tissue-specific TWAS analysis of 211 neuroimaging traits (n=19,629 subjects for ROI volumes and 17,706 for DTI parameters). The p-values are raw p-values of two-sided Generalized BerkJones (GBJ) test.

File Name: Supplementary Data 13
Description: Enrichment analysis of genes identified for 211 neuroimaging traits (n=19,629 subjects for ROI volumes and 17,706 for DTI parameters). The p-values are raw p-values of two-sided Wilcoxon rank test.

File Name: Supplementary Data 14
Description: List of cross-tissue TWAS-significant gene-trait associations that have larger than 0.5 regional colocalization probability (RCP) in ENLOC colocalization analysis (n=19,629 subjects for ROI volumes and 17,706 for DTI parameters.

File Name: Supplementary Data 15
Description: Sources of the 16 sets of publicly available GWAS summary statistics of other complex traits and clinical outcomes used in this study.

File Name: Supplementary Data 16
Description: List of significant gene-trait associations discovered in UKB cross-tissue TWAS analysis of 16 other complex traits and clinical outcomes. The p-values are raw p-values of two-sided Generalized Berk- Jones (GBJ) test.

File Name: Supplementary Data 17
Description: Prediction accuracy (incremental R-squared) and pvalue of gene-based polygenic risk scores constructed by UKB-derived TWAS summary statistics (n=19,629 subjects) on the four independent datasets. The p-values are asymptotic p-values of two-sided t-test statistics in linear regression.

File Name: Supplementary Data 18
Description: Prediction accuracy (incremental R-squared) and pvalue of gene-based polygenic risk scores constructed by UKB-derived GWAS summary statistics (n=17,706 subjects) on the three independent datasets. The p-values are asymptotic p-values of two-sided t-test statistics in linear regression.

File Name: Supplementary Data 19
Description: Prediction accuracy (incremental R-squared) of gene-based polygenic risk scores (PRS) constructed by UKB-derived TWAS summary statistics conditioning on variant-based PRS constructed by UKBderived GWAS summary statistics (n=19,629 subjects) on the four independent datasets.

File Name: Supplementary Data 20
Description: Prediction accuracy (incremental R-squared) of combining gene-based polygenic risk scores (PRS) constructed by UKBderived TWAS summary statistics and variant-based PRS constructed by UKB-derived GWAS summary statistics (n=19,629 subjects) on the four independent datasets.

File Name: Supplementary Data 21
Description: Prediction accuracy (incremental R-squared) of variant-based PRS constructed by UKB-derived GWAS summary statistics conditioning on gene-based polygenic risk scores (PRS) constructed by UKBderived TWAS summary statistics (n=19,629 subjects) on the four independent datasets.

File Name: Supplementary Data 22
Description: Mean prediction accuracy (incremental R-squared) of gene-based polygenic risk scores constructed using each reference panel (n=19,629 subjects for ROI volumes and 17,706 for DTI parameters).

File Name: Supplementary Data 23
Description: Sample size of each neuroimaging GWAS and IDs of the 211 neuroimaging traits (101 ROI volumes and 110 DTI parameters).
