# Supplementary material for: Transcriptome-wide association analysis of brain structures yields insights into pleiotropy with complex neuropsychiatric traits
Source: Nat Commun. 2021 May 17;12:2878. doi: 10.1038/s41467-021-23130-y (PMC8128893; doi:10.1038/s41467-021-23130-y)
Supplement: Supplementary file 5 — Reporting Summary [file 41467_2021_23130_MOESM5_ESM.pdf]

nature research

Corresponding author(s): Yun Li, Hongtu Zhu

Last updated by author(s): Mar 25, 2021

Reporting Summary

Nature Research wishes to improve the reproducibility of the work that we publish. This form provides structure for consistency and transparency in reporting. For further information on Nature Research policies, see Authors & Referees and the Editorial Policy Checklist .

Statistics

For all statistical analyses, confirm that the following items are present in the figure legend, table legend, main text, or Methods section.

n/a	Confirmed	
☐	☒	The exact sample size (n) for each experimental group/condition, given as a discrete number and unit of measurement
☐	☒	A statement on whether measurements were taken from distinct samples or whether the same sample was measured repeatedly
☐	☒	The statistical test(s) used AND whether they are one- or two-sided<br>*Only common tests should be described solely by name; describe more complex techniques in the Methods section.*
☐	☒	A description of all covariates tested
☐	☒	A description of any assumptions or corrections, such as tests of normality and adjustment for multiple comparisons
☐	☒	A full description of the statistical parameters including central tendency (e.g. means) or other basic estimates (e.g. regression coefficient) AND variation (e.g. standard deviation) or associated estimates of uncertainty (e.g. confidence intervals)
☐	☒	For null hypothesis testing, the test statistic (e.g. F, t, r) with confidence intervals, effect sizes, degrees of freedom and P value noted<br>*Give P values as exact values whenever suitable.*
☐	☒	For Bayesian analysis, information on the choice of priors and Markov chain Monte Carlo settings
☐	☒	For hierarchical and complex designs, identification of the appropriate level for tests and full reporting of outcomes
☐	☒	Estimates of effect sizes (e.g. Cohen's d, Pearson's r), indicating how they were calculated

Our web collection on statistics for biologists contains articles on many of the points above.

Software and code

Policy information about availability of computer code

Data collection	UTMOST V2018-04-24, https://github.com/Joker-Jerome/UTMOST;<br>FUSION, http://gusevlab.org/projects/fusion/;<br>PLINK v1.90 beta, https://www.cog-genomics.org/plink2/;<br>R (version 3.5.0)
Data analysis	UTMOST V2018-04-24, https://github.com/Joker-Jerome/UTMOST;<br>FUSION, http://gusevlab.org/projects/fusion/;<br>R (version 3.5.0)

For manuscripts utilizing custom algorithms or software that are central to the research but not yet described in published literature, software must be made available to editors/reviewers. We strongly encourage code deposition in a community repository (e.g. GitHub). See the Nature Research guidelines for submitting code & software for further information.

Data

Policy information about availability of data

All manuscripts must include a data availability statement. This statement should provide the following information, where applicable:

- Accession codes, unique identifiers, or web links for publicly available datasets
- A list of figures that have associated raw data
- A description of any restrictions on data availability

The data used in this work was obtained from publicly available datasets: the UK Biobank (UKB) study, the Human Connectome Project (HCP) study, the Pediatric Imaging, Neurocognition, and Genetics (PING) study, the Philadelphia Neurodevelopmental Cohort (PNC) study, the Alzheimer's Disease Neuroimaging Initiative (ADNI) study, and ENIGMA2 & the ENIGMA-CHARGE collaboration. For the first five datasets, the raw MRI, covariates and SNP data were available from each data resource:
UK Biobank, http://www.ukbiobank.ac.uk/resources/;

PING, http://pingstudy.ucsd.edu/resources/genomics-core.html/;
PNC, https://www.ncbi.nlm.nih.gov/projects/gap/cgi-bin/study.cgi?study_id=phs000607.v1.p1/;
ADNI, http://adni.loni.usc.edu/data-samples/; and
HCP, https://www.humanconnectome.org/.

The GWAS summary statistics can be obtained at https://github.com/BIG-S2/GWAS and http://enigma.ini.usc.edu/research/. In addition, we used other 16 sets of publicly available GWAS summary statistics shared by several GWAS databases. These data resources were summarized in Supplementary Data 15. The FUSION database used in this study is available at http://gusevlab.org/projects/fusion/.

Field-specific reporting

Please select the one below that is the best fit for your research. If you are not sure, read the appropriate sections before making your selection.

[x] Life sciences   [ ] Behavioural & social sciences   [ ] Ecological, evolutionary & environmental sciences

For a reference copy of the document with all sections, see nature.com/documents/nr-reporting-summary-flat.pdf

Life sciences study design

All studies must disclose on these points even when the disclosure is negative.

Sample size

No power calculation was needed in advance. The previous GWAS used all samples passing standard quality controls (please see below). And we used all the GWAS summary statistics that are available from these GWAS.

Data exclusions

Full details of the data quantity controls steps of these GWAS can be found in the Online Methods section and/or Supplementary Note of Zhao et al. (https://doi.org/10.1038/s41588-019-0516-6), Zhao et al. (https://doi.org/10.1038/s41380-019-0569-z), Hibar et al. (https://doi.org/10.1038/nature14101), and Adams et al. (https://doi.org/10.1038/nn.4398). In these GWAS, the UKB discovery study made use of individuals of British ancestry from the UKB study, and the other five sets GWAS were performed on individuals of European ancestry. No data that passing the above pre-established quantity controls were excluded from the analyses.

Replication

For discovery, we used the GWAS summary statistics of the UKB study. Then the GWAS results of the other five studies were used for validation.

Randomization

The datasets are from observational studies which did not include randomized experiments. Therefore, randomization is not relevant to the study.

Blinding

The datasets are from observational studies and the current study did not include an experimental design, thus there is no step equivalent to blinding involved.

Reporting for specific materials, systems and methods

We require information from authors about some types of materials, experimental systems and methods used in many studies. Here, indicate whether each material, system or method listed is relevant to your study. If you are not sure if a list item applies to your research, read the appropriate section before selecting a response.

Materials & experimental systems

n/a	Involved in the study	
[x]	[ ]	Antibodies
[x]	[ ]	Eukaryotic cell lines
[x]	[ ]	Palaeontology
[x]	[ ]	Animals and other organisms
[ ]	[x]	Human research participants
[x]	[ ]	Clinical data

Methods

n/a	Involved in the study	
[x]	[ ]	ChIP-seq
[x]	[ ]	Flow cytometry
[ ]	[x]	MRI-based neuroimaging

Human research participants

Policy information about studies involving human research participants

Population characteristics	The main GWAS made use of data of individuals of British ancestry from the UKB study, and the other five GWAS were performed on individuals of European ancestry. Particularly, the UKB genetic data ~8m SNPs after genotyping quality controls, all individuals were ages between 40 and 80 with mean 62.51, the proportion of male is 0.47. See Supplementary Data 23 for a summary of sample size and the analyzed neuroimaging traits of each GWAS.
Recruitment	Recruitment details and dataset overviews can be found in https://doi.org/10.1371/journal.pmed.1001779 for UKB, https://doi.org/10.1016/j.neuroimage.2013.07.064 for PNC, https://doi.org/10.1016/j.jalz.2013.05.1769 for ADNI, https://doi.org/10.1016/j.neuroimage.2015.04.057 for PING, and https://doi.org/10.1038/nature14101 and https://doi.org/10.1038/nn.4398 for ENIGMA. We are not aware of recruitment biases that are likely to have a major impact on the results in the current study.
Ethics oversight	The data resources had obtained informed consent from all participants and had obtained approval from their research ethics committees or institutional review boards. The UKB study had obtained ethics approval from the North West Multicentre Research Ethics Committee (approval number: 11/NW/0382). ADNI study was approved by all the institutional ethical review boards of all participating centers. The institutional review boards of the University of Pennsylvania and the Children's Hospital of Philadelphia approved all study procedures in the PNC study. The human research protection programs and institutional review boards at the nine institutions participating in the PING project approved all experimental and consenting procedures. All experimental procedures in the HCP study were approved by the institutional review boards at Washington University (approval number: 201204036).

Note that full information on the approval of the study protocol must also be provided in the manuscript.

Magnetic resonance imaging

Experimental design

Design type	This study made use of imaging data from Structural MRI, Diffusion MRI, and genetic SNP data.
Design specifications	Details can be found in Miller et al. (doi:10.1038/nn.4393) and Alfaro-Almagro et al. (https://doi.org/10.1016/j.neuroimage.2017.10.034) for UKB, https://doi.org/10.1016/j.neuroimage.2013.07.064 for PNC, https://doi.org/10.1016/j.jalz.2013.05.1769 for ADNI, https://doi.org/10.1016/j.neuroimage.2015.04.057 for PING, and https://doi.org/10.1038/nature14101 and https://doi.org/10.1038/nn.4398 for ENIGMA. Data procession can be found in previous GWAS, including Zhao et al. (https://doi.org/10.1038/s41588-019-0516-6), Zhao et al. (https://doi.org/10.1038/s41380-019-0569-z), Hibar et al. (https://doi.org/10.1038/nature14101), and Adams et al. (https://doi.org/10.1038/nn.4398).
Behavioral performance measures	Behavioral performance measures were not used in this study.

Acquisition

Imaging type(s)	Structural and Diffusion
Field strength	3T in UKB, PNC, PING, and HCP; 1.5 T or 3T for ADNI. The ENIGMA data were collected at all participating sites around the world, so the field strength can be different from each other.
Sequence & imaging parameters	Details can be found in Miller et al. (doi:10.1038/nn.4393) and Alfaro-Almagro et al. (https://doi.org/10.1016/j.neuroimage.2017.10.034) for UKB, https://doi.org/10.1016/j.neuroimage.2013.07.064 for PNC, https://doi.org/10.1016/j.jalz.2013.05.1769 for ADNI, https://doi.org/10.1016/j.neuroimage.2015.04.057 for PING, and https://doi.org/10.1038/nature14101 and https://doi.org/10.1038/nn.4398 for ENIGMA.
Area of acquisition	The whole brain scan was used.

Diffusion MRI ☒ Used ☐ Not used

Parameters	For each of the two diffusion-weighted shells, 50 distinct diffusion-encoding directions were acquired, two b-values (b = 1,000 and 2,000 s/mm2) are used, and cardiac gating was not used.

Preprocessing

Preprocessing software	Details can be found in Miller et al. (doi:10.1038/nn.4393) and Alfaro-Almagro et al. (https://doi.org/10.1016/j.neuroimage.2017.10.034) for UKB, https://doi.org/10.1016/j.neuroimage.2013.07.064 for PNC, https://doi.org/10.1016/j.jalz.2013.05.1769 for ADNI, https://doi.org/10.1016/j.neuroimage.2015.04.057 for PING, and https://doi.org/10.1038/nature14101 and https://doi.org/10.1038/nn.4398 for ENIGMA.
Normalization	Details can be found in Miller et al. (doi:10.1038/nn.4393) and Alfaro-Almagro et al. (https://doi.org/10.1016/j.neuroimage.2017.10.034) for UKB, https://doi.org/10.1016/j.neuroimage.2013.07.064 for PNC, https://doi.org/10.1016/j.jalz.2013.05.1769 for ADNI, https://doi.org/10.1016/j.neuroimage.2015.04.057 for PING, and https://doi.org/10.1038/nature14101 and https://doi.org/10.1038/nn.4398 for ENIGMA.

Normalization template

Details can be found in Miller et al. (doi:10.1038/nn.4393) and Alfaro-Almagro et al. (https://doi.org/10.1016/j.neuroimage.2017.10.034) for UKB, https://doi.org/10.1016/j.neuroimage.2013.07.064 for PNC, https://doi.org/10.1016/j.jalz.2013.05.1769 for ADNI, https://doi.org/10.1016/j.neuroimage.2015.04.057 for PING, and https://doi.org/10.1038/nature14101 and https://doi.org/10.1038/nn.4398 for ENIGMA.

Noise and artifact removal

Details can be found in Miller et al. (doi:10.1038/nn.4393) and Alfaro-Almagro et al. (https://doi.org/10.1016/j.neuroimage.2017.10.034) for UKB, https://doi.org/10.1016/j.neuroimage.2013.07.064 for PNC, https://doi.org/10.1016/j.jalz.2013.05.1769 for ADNI, https://doi.org/10.1016/j.neuroimage.2015.04.057 for PING, and https://doi.org/10.1038/nature14101 and https://doi.org/10.1038/nn.4398 for ENIGMA.

Volume censoring

Details can be found in Miller et al. (doi:10.1038/nn.4393) and Alfaro-Almagro et al. (https://doi.org/10.1016/j.neuroimage.2017.10.034) for UKB, https://doi.org/10.1016/j.neuroimage.2013.07.064 for PNC, https://doi.org/10.1016/j.jalz.2013.05.1769 for ADNI, https://doi.org/10.1016/j.neuroimage.2015.04.057 for PING, and https://doi.org/10.1038/nature14101 and https://doi.org/10.1038/nn.4398 for ENIGMA.

Statistical modeling & inference

Model type and settings

Details can be found in Miller et al. (doi:10.1038/nn.4393) and Alfaro-Almagro et al. (https://doi.org/10.1016/j.neuroimage.2017.10.034) for UKB, https://doi.org/10.1016/j.neuroimage.2013.07.064 for PNC, https://doi.org/10.1016/j.jalz.2013.05.1769 for ADNI, https://doi.org/10.1016/j.neuroimage.2015.04.057 for PING, and https://doi.org/10.1038/nature14101 and https://doi.org/10.1038/nn.4398 for ENIGMA.

Effect(s) tested

Details can be found in Miller et al. (doi:10.1038/nn.4393) and Alfaro-Almagro et al. (https://doi.org/10.1016/j.neuroimage.2017.10.034) for UKB, https://doi.org/10.1016/j.neuroimage.2013.07.064 for PNC, https://doi.org/10.1016/j.jalz.2013.05.1769 for ADNI, https://doi.org/10.1016/j.neuroimage.2015.04.057 for PING, and https://doi.org/10.1038/nature14101 and https://doi.org/10.1038/nn.4398 for ENIGMA.

Specify type of analysis: ☐ Whole brain ☐ ROI-based ☒ Both

Anatomical location(s)

Details can be found in https://doi.org/10.1016/j.neuroimage.2010.09.025 and https://doi.org/10.1016/j.neuroimage.2013.04.061.

Statistic type for inference
(See Eklund et al. 2016)

Inference was not carried out when generating imaging phenotypes.

Correction

Inference was not carried out when generating imaging phenotypes.

Models & analysis

n/a	Involved in the study
☒	☐ Functional and/or effective connectivity
☒	☐ Graph analysis
☒	☐ Multivariate modeling or predictive analysis
